# Elastic Entropic Forces in Polymer Deformation

**DOI:** 10.3390/e24091260

**Published:** 2022-09-07

**Authors:** Vladimir I. Kartsovnik, Dimitri Volchenkov

**Affiliations:** 1 Gesellschaft für Kultur, Igenieurwesen und Wissenschaften e.V., Bautzner Str. 20 HH, 01099 Dresden, Germany; 2 Department of Mathematics and Statistics, Texas Tech University, 1108 Memorial Circle, Lubbock, TX 79409, USA

**Keywords:** entropic elastic force in macromolecules, viscosity activation energy, viscosity anomaly, standard linear solid model, Kelvin–Voigt model, creep prediction, hysteresis in rubber, 36.20.Ey, 62.20.Dc, 62.20.Fe, 62.20.Hg, 81.40.Lm

## Abstract

The entropic nature of elasticity of long molecular chains and reticulated materials is discussed concerning the analysis of flows of polymer melts and elastomer deformation in the framework of Frenkel–Eyring molecular kinetic theory. Deformation curves are calculated in line with the simple viscoelasticity models where the activation energy of viscous flow depends on the magnitude of elastic entropic forces of the stretched macromolecules. The interconnections between deformation processes and the structure of elastomer networks, as well as their mutual influence on each other, are considered.

## 1. Introduction

In the vacancy mechanism of self-diffusion in liquid bodies and deformation of solids proposed in the early works of Frenkel [1] and Eyring [2,3], a diffusing atom, or a molecule, moves into neighboring vacancies by jumping due to thermal motion. This process is described macroscopically by Hooke’s law, relating elastic stresses σ to strains ε, viz.,

(1) σ=Eε

where *E* is the Young’s modulus describing the relative stiffness of a material, which is measured by the slope of elastic of a stress and strain graph, and by Newton’s flow law,

(2) $$\tau \;\;=\;\;\eta \;\dot{\gamma },$$

stating that the application of shear stress τ on a liquid leads to the share rate $\dot{\gamma }$ in direct proportion to the amount of stress applied, with the coefficient of (apparent) viscosity η. Eyring’s absolute rate theory [3], used in chemical kinetics to describe changes in the rate of chemical reactions against absolute temperature $k_{B}T$, predicts the following dependence of dynamic viscosity on shear stress [3,4,5,6,7], viz.,

(3) $$\eta \;\;=\;\;B\tau \exp\left(\frac{E_{0}-b\tau }{k_{B}T}\right)$$

where $E_{0}$ is the activation energy required for a molecule to jump, *B* is a pre-exponential factor, *b* is Eyring’s coefficient of viscous volume [3], and $k_{B}$ is Boltzmann’s constant. Although most low molecular weight liquids do obey simple Newton’s law (2), the anomalous behavior of viscosity following from Eyring’s equation (Equation (3)) was observed experimentally in the studies of deformation process [8] where shear stress transferred through a system of elastic interatomic bonds may act directly on atoms during their thermal motion. Viscosity anomalies manifesting themselves in the form of decreasing viscosity against increasing shear rate and shear stress are well known as well [9,10]. However, when expanding the range of shear rates in experiments with polymers, the observed flow curves may significantly deviate from those predicted by Eyring’s law (3), and feature another form of viscosity anomaly. Many attempts have been made to generalize Eyring’s formula for the different types of *molecular kinetic units* (MKU) [11] and in various viscous liquid flows rising the numerous variants of empirical rheological equations in the literature [9,12]. The profound limitation of Eyring’s equation (Equation (3)) in its application to the flows of polymer melts is that it does not account for a possible influence of reversible, rubber-like deformations of MKU stretched along the flow [5,13,14].

In our paper, we review and analyze another explanation for viscosity anomalies in polymer flows, taking into account the entropic nature of elasticity emerging in macromolecules. In our approach, activation energy $E_{0}$ in Eyring’s equation (Equation (3)) takes into account moving of an entire macromolecule with its valence bonds instead of a jump of a single atom in thermal motion [14,15]. While in the flow of polymer melts, the macromolecule can jump over the potential barrier and take a new equilibrium conformation, maintaining the chemical structure of the kinetic unit. Furthermore, the macromolecule might be stretched along the gradient of flow rate due to external forces acting in the fluid flow [16,17,18].

Statistical theory of rubber elasticity [19,20] states that stretching would excite the *entropic elastic forces* (EEF) acting along the stretched macromolecular chains. For example, the rotor of a viscometer immersed into polymer melts rotates backward after the engine shuts down, testifying to highly elastic reversible deformation present in the flow of polymer melts [9,21]. The experimentally observed rubber elasticity increase as the temperature rises also confirms the entropic nature of elasticity [19,22,23,24], indicating that the kinetic effects of catenation and entanglements of molecular chains should be taken into account while modeling deformation and flowing processes in melted polymers [25,26,27,28,29].

In Section 2, we analyzed a possible effect of chain and network structures of the polymer melts and elastomers on their kinematic and mechanical properties. Namely, in Section 2.1, we discuss how stretching a polymer chain in flowing polymer melts may result in emerging viscosity anomalies due to the effect of entropic elastic forces reducing the activation energy. In Section 2.2, we discuss an opposite effect when the action of EEF might increase the activation energy of jumps in elastomer networks. The effect of these forces in creeping behavior is discussed in Section 2.3. The next section, Section 3, is devoted to the experimental verification of relations manifesting the effect of EEF discussed theoretically in Section 2. Namely, in Section 3.1, we discuss the experiments exposing the entropic nature of viscosity anomaly in the flow of polystyrene. The rubber stretching experiments revealing a hysteresis effect (the Mullins effect) due to the action of EEF emerging in deforming elastomers are reported in Section 3.2. An experimental study of rubber creeping behavior is reported in Section 3.3. In Section 4, we summarize the experimental observation on the effect of EEF on polymer’s deformation. We conclude in the last section.

## 2. Methods: Accounting for Entropic Elastic Forces in Polymer Deformation Processes

In the present section, we discuss a possible change in the jump activation energy fostered by the effect of EEF reflecting the molecular structure of polymers. The chain and network molecular structure of polymers manifests in viscosity anomalies registered in polymer flows, a hysteresis phenomenon of tensile curves in elastomers, and the creeping behavior in silicon rubber.

### 2.1. Viscosity Anomalies in Polymer Flows

Stretching polymer chains from an equilibrium conformation in flowing polymer melts results in manifestation of the EEF $f_{e}$ [19,22,23] in proportion to the distance, r, between the ends of a macromolecule (see Appendix A), viz.,

(4) $$f_{e}=\frac{3k_{B}T}{Nl^{2}}r$$

where *N* is the number of monomeric links in the chain, and *l* is the length of each unit. The emerging entropic force (4) reduces activation energy $E_{0}$ allowing for the MKU to jump by a distance λ proportional to the magnitude of EEF in the direction of flow forces [5,7,14,15,18], viz.,

(5) $$\eta \;\;=\;\;B\tau \exp\left(\frac{E_{0}-\delta \gamma_{e}}{k_{B}T}\right),\;\delta =\frac{1}{2}\lambda \frac{3k_{B}T}{Nl^{2}}a$$

where *B* is a pre-exponential factor, δ is an activation coefficient having the dimension of energy, *a* is a coefficient having the dimension of length, and $\gamma_{e}$ is a dimensionless size of reversible, rubber-like deformations emerging due to chemical bonding between the monomeric links of a macromolecule [21,23,26]. The value $\gamma_{e}$ can be assessed by measuring the volume of recoverable deformation in polymer melts after stopping the rotary plane (cone) in a viscometer [14]. Plugging the expression (5) back into Newton’s formula (2) and applying the logarithm to the resulting equation, we obtain three linear relationships between the logarithms of viscosity η, shear stress τ, and share rate $\dot{\gamma }$ that might be verified experimentally (see Section 3.1), viz.,

(6) $$\ln\eta \;\;=\;\;\left[\ln\tau -\ln\frac{1}{B}+\frac{E_{0}}{k_{B}T}\right]-\frac{\delta }{k_{B}T}\gamma_{e},$$

(7) $$\ln\dot{\gamma }\;\;=\;\;\left[\ln\frac{1}{B}-\frac{E_{0}}{k_{B}T}\right]+\frac{\delta }{k_{B}T}\gamma_{e},$$

(8) $$\ln\tau \;\;=\;\;\left[\ln\eta +\ln\frac{1}{B}-\frac{E_{0}}{k_{B}T}\right]+\frac{\delta }{k_{B}T}\gamma_{e},$$

indicating that anomalous viscosity might be considered a manifestation of the EEF reducing jump activation energy in the flow of polymer melts [5,14,15].

### 2.2. Dependence of Elastomer Tensile Curves on Elastic Entropic Forces

In Section 2.1, we discussed that the EEF, $f_{e},$ emerging in the flow of polymer melts reduces the activation free energy $E_{0}$ required for the MKU to jump in the direction of external forces by a magnitude proportional to $f_{e}$. However, the EEF may cause an opposite effect as well, *increasing* the activation energy of jumps in elastomeric networks [5,30].

In the temperature range above the glass transition, the polymer structure turns viscous, amorphous, or rubbery, so that polymers can be considered as elastomers [22,23]. Special features of deformation processes in elastomers, both in glassy and highly elastic amorphous states, are associated with their network molecular structures [23,27]. The most prominent features of the stretching process in elastomers are the accelerated stress growth up to breaking elongations of material and large elastic deformations self-reversing after removing the force or load [28,29]. The ability of elastomers to sustain high reversible deformations is associated with the entropic nature of the stretching behavior of long macromolecules in a condensed amorphous state [20,30].

Statistical rubber elasticity theory [19,25,31,32,33,34,35,36,37,38,39,40], elaborated in its application to elastomer networks, sets out the dependence of the measured stress specific to the initial cross-section of a sample, $\sigma_{m}$, and the specimen current extension ratio, $\lambda =l/l_{0}$, with respect to its initial length $l_{0},$ in the following form:

(9) $$\sigma_{m}\;\;=\;\;E\left(\lambda -\frac{1}{\lambda^{2}}\right),\;E\;\;=\;\;g\frac{\rho RT}{M_{c}}\left(1-\frac{2M_{c}}{M}\right)\left(2\lambda^{2}+\frac{1}{\lambda }\right)$$

where *R* is the gas constant, *T* is the absolute temperature, *E* is the isothermal Young’s modulus, $M_{c}$ is the molecular weight of a chain segment characterizing the number of chemical cross-links in the network, *M* is the initial molecular weight of the elastomer before cross-linking, ρ is the density of the rubber, and *g* is Flory’s correction factor accounting for the network structural flaws that may affect its deformation behavior [40,41,42].

**Figure 1:**
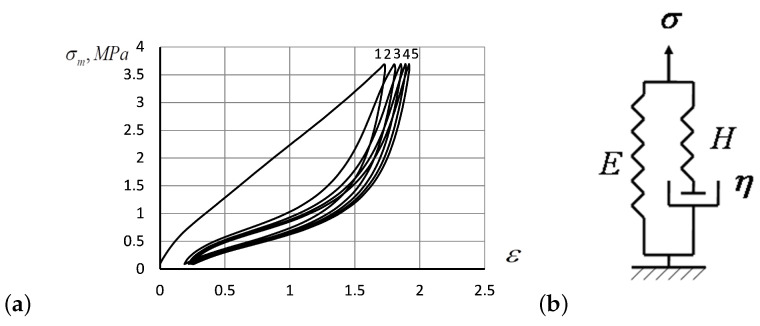
(**a**) The hysteresis curves for a PMVS rubber specimen subjected to repeated forced extension, and compression at 250 mm/min at room temperature, with the relative strain ε. (**b**) The *standard linear solid* (SLS) mechanical model.

Empirically observed tensile curves for rubber fit the behavior predicted by the theoretical model (9) only for the uniaxial extensions of λ≈1. Other types of dependence, including many empirical parameters, were discussed in the literature [43] and designed to fit the experimental tensile curves better at high elongations. Furthermore, stretching-induced structural changes in rubber reveal themselves in the effect of mechanical hysteresis (Mullins’ effect): after a rubber specimen is extended for the first time and then allowed to recover from the deformation, repeated extension by the same amount requires significantly lower stress values [28,29,44]. In Figure 1a, we have shown the hysteresis curves experimentally observed by us in silicone rubber *poly*(*methylvinylsiloxane*) (PMVS) subjected to tensile deformation against the relative strain ε, at 250 mm/min until the stress reached 3.8 MPa. After stretching, the specimen was subjected to the reverse deformation, until the stress had been completely relaxed [30]. The deformation rounds were then repeated four times. The first ascending tensile curve shifted toward larger strain values in Figure 1a indicates the significant softening of the material due to rupture of physical cross-links during the first round of deformation [5,30].

Before explaining the Mullins effect, let us consider the *standard linear solid* (SLS) model schematically presented in Figure 1b first. Accordingly the SLS model, the external stress, σ, applied to a specimen sums of two components: (i.) the stress $\sigma_{e}$ on the spring of stiffness *E*, and (ii.) the viscous stress $\sigma_{\eta }$ manifested by the elastic spring connected in series with the dashpot in Figure 1b with Hooke’s modulus *H* associated with the viscous element η. Assuming that the stress $\sigma_{e}$ takes the form of Hooke’s law (1), $\sigma_{e}=E\varepsilon ,$ we may express the viscous stress as $\sigma_{\eta }=\sigma -E\varepsilon$ accordingly the SLS model.

The differential equation for the SLS model [45], expressing the dynamics of stress–strain relation for rubber at constant tensile strain rate, $\dot{\varepsilon }=\mathrm{const},$ and constant dashpot viscosity,

(10) $$\eta_{0}\;\;=\;\;A\exp\left(\frac{E_{0}}{k_{B}T}\right)\;\;=\;\;\mathrm{const}$$

where *A* is some pre-exponential factor, independent of the applied stress σ, and can be written as follows:

(11) $$\eta_{0}\left[\left(H+E\right)\dot{\varepsilon }-\dot{\sigma }\right]=H\left(\sigma -E\varepsilon \right).$$

As the strain grows linearly over time, $\varepsilon \left(t\right)=\varepsilon_{0}+\dot{\varepsilon }t$, with a constant strain rate $\dot{\varepsilon }$, the general solution of the SLS Equation (11) takes the following form:

(12) $$\sigma_{SLS}\left(t\right)\;\;=\;\;E\underset{\varepsilon (t)}{\underset{︸}{\left(\varepsilon_{0}+\dot{\varepsilon }t\right)}}+\eta_{0}\dot{\varepsilon }+\exp\left(-\frac{t}{\eta /H}\right)$$

approaching a linear stress–strain relation over the characteristic relaxation time η/H. The SLS model predicts (12) that when a specimen is stretched at a constant rate, the growth of strain-dependent stress will gradually slow down and evolve into the linear stress–strain dependence, as t≫η/H, viz.,

(13) $$\sigma_{SLS}\left(\varepsilon \right)=E\varepsilon +\eta_{0}\dot{\varepsilon }.$$

The linear SLS model does not explain the hysteresis phenomena.

The hysteresis behavior in elastomers is entirely due to the action of EEF emerging in the molecular network undergoing deformation. Namely,

(1) With *increasing* deformation over the stretching phase, the EEF *reinforces* the resistance of molecular network against the tensile stress σ, fostering an *increase* of the jump activation energy $E_{0}$ in Eyring’s formula (3) by an amount $b_{S}\sigma_{\eta }$ where $b_{S}$ is a viscous volume coefficient related to the stretching deformation $\sigma_{\eta }$ [5,14,30], viz.,
  (14) $$\eta_{S}=A_{S}\sigma_{\eta }\exp\left(\frac{E_{0}+b_{S}\sigma_{\eta }}{k_{B}T}\right)=A_{S}\left(\sigma -E\varepsilon \right)\exp\left(\frac{E_{0}+b_{S}\left(\sigma -E\varepsilon \right)}{k_{B}T}\right)$$
  where $A_{S}$ is the pre-exponential factor calculated by absolute reaction rate theory, and the index *S* pertains to the stretching phase of deformation.
(2) In a retraction phase of deformation, the EEF *decreases* the jump activation energy $E_{0}$ by an amount $b_{R}\sigma_{\eta }$, as the action of EEF *coincides* with the retraction direction of the specimen deformation, and, therefore,
  (15) $$\eta_{R}=A_{R}\left(\sigma -E\varepsilon \right)\exp\left(\frac{E_{0}-b_{R}\left(\sigma -E\varepsilon \right)}{k_{B}T}\right)$$
  where the corresponding pre-exponential factor $A_{R}$ and viscous volume coefficient $b_{R}$ are indexed by R, being related to the *retraction* phase of deformation.

By equating the expression for viscosity following from the SLS model (11) to Eyring’s viscosity accounting, for the effect of EEF in the course of deformation (14–15), we obtain the following equation introducing the action of EEF into the stress–strain dynamics:

(16) $$\frac{H(\sigma -E\varepsilon )}{\left(H+E\right)\dot{\varepsilon }-\dot{\sigma }}=A_{S/R}\left(\sigma -E\varepsilon \right)\exp\left(\frac{E_{0}\pm b_{S/R}\left(\sigma -E\varepsilon \right)}{k_{B}T}\right),$$

or, after dividing both sides of the latter equation by (σ−Eε) and applying the logarithm on them, viz.,

(17) $$-\ln\left(\left(H+E\right)\dot{\varepsilon }-\dot{\sigma }\right)=\ln\left(\frac{\eta_{0}}{H}\right)\pm \frac{b_{S/R}}{k_{B}T}\left(\sigma -E\varepsilon \right).$$

As the first term in the right-hand side of (17) is constant for a given material and as $\dot{\varepsilon }=\mathrm{const}$, the latter equation predicts the *linear* relation between the quantity in the left-hand side of (17), −$\ln\left(\left(H+E\right)\dot{\varepsilon }-\dot{\sigma }\right),$ and the viscous stress (σ−Eε) that might be verified experimentally (see Section 3.2 for details).

**Figure 2:**
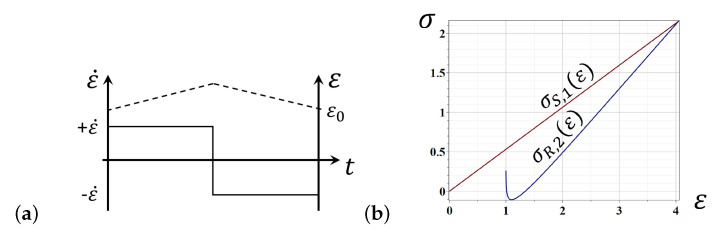
(**a**) The strain dynamics in the case of constant strain rate $\pm \dot{\varepsilon }$ over the stretching and retracting deformations. (**b**) The model stress–strain curves for the constant strain rate $\pm \dot{\varepsilon }$ over the stretching and retracting deformations.

As the strain function in (17) changes linearly over time, viz., $\varepsilon \left(t\right)=\varepsilon_{0}+\dot{\varepsilon }t,$ during the stretching phase, and $\varepsilon \left(t\right)=\varepsilon_{\max}-\dot{\varepsilon }t,$ during the retraction phase of deformation (Figure 2a), the general solutions for Equation (17) can be calculated analytically. Namely, in the stretching phase, these solutions are as follows:

(18) $$\sigma_{S,1}\left(\varepsilon \right)\;\;=\;\;E\varepsilon -\frac{k_{B}T}{b_{S}}\ln\left(\eta_{0}\dot{\varepsilon }\right)$$

(19) $$\sigma_{S,2}\left(\varepsilon \right)\;\;=\;\;E\varepsilon -\frac{k_{B}T}{b_{S}}\ln\left(\frac{\eta_{0}\dot{\varepsilon }}{1-\exp\left(\frac{Hb_{S}}{k_{B}T}\left(\varepsilon -\varepsilon_{0}\right)\right)}\right),\;\varepsilon_{0}<\varepsilon .$$

The first solution (18) predicts a linear stress–strain relation in the stretching phase, similar to that of the SLS model (13). The second solution (19) does not exist for $\varepsilon >\varepsilon_{0}$ (as producing a negative number under the logarithm), being non-physical, at least, for the first stretching move: the first tensile curve describing the initial specimen stretching is linear. However, the solution (19) may become real in the further stretching rounds, as seen in the forthcoming hysteresis loops (Figure 1a) whenever strain is decreasing, $\varepsilon <\varepsilon_{0}$.

In the retraction phase, the Equation (17) has only one admissible solution, as the formal, linear solution, $\sigma_{R,1}\left(\varepsilon \right)=E\varepsilon +k_{B}T/b_{S}\times \ln\left(-\eta_{0}\dot{\varepsilon }\right),$ does not exist (due to a negative number under the logarithm): there is no linear stress–strain relation under retraction possible. However, the second solution exists in the retraction phase, viz.,

(20) $$\sigma_{R,2}\left(\varepsilon \right)\;\;=\;\;E\varepsilon +\frac{k_{B}T}{b_{S}}\ln\left(\frac{\eta_{0}\dot{\varepsilon }}{\exp\left(\frac{Hb_{S}}{k_{B}T}\left(\varepsilon_{0}-\varepsilon \right)\right)-1}\right),\;\varepsilon_{0}>\varepsilon .$$

The modeling curves representing the analytic solutions (18) and (20) in the first stretching–retraction round of deformation are shown in Figure 2b.

The analytical solution of the modified SLS model taking into account the EEF effect predicts a linear stress–strain relation for the first round of specimen stretching, followed by the non-linear tensile curves for the further rounds of deformation.

The proposed model accounting for the effect of the EEF on elastomer deformation predicts the linear relation between the value $\ln(\left(H+E\right)\dot{\varepsilon }-\dot{\sigma })$ and (σ−Eε) that might be verified experimentally (see Section 3.2).

### 2.3. Entropic Elastic Forces for Creeping Prediction

Creeping reflects the tendency of a material to deform plastically over time at any level of compressive, tensile, and shear mechanical stress applied. The creep behavior of elastomers subjected to the total stress σ can be described by the simple *Kelvin–Voigt model* (KVM), viz.,

(21) $$\sigma \;\;=\;\;\sigma_{e}+\sigma_{\eta }\;\;=\;\;E\varepsilon +\eta \dot{\varepsilon }$$

where $\sigma_{e}=E\varepsilon$ is the elastic component of stress (1), ε is strain, $\dot{\varepsilon }$ is the strain rate, and $\sigma_{\eta }=\eta \dot{\varepsilon }$ represents stress on the dashpot according to Newton’s law (2). Expressing the viscosity parameter from (21) as $\eta =\left(\sigma -E\varepsilon \right)/\dot{\varepsilon }$, equating it to the viscosity equation for a stretching phase (14), and dividing the resulting equation by the common factor (σ−Eε), we obtain the *Kelvin–Voigt differential equation* (KVDE) describing strain dynamics in the stretching phase, viz.,

(22) $$\frac{1}{\dot{\varepsilon }}\;\;=\;\;A_{S}\exp\left(\frac{E_{0}+b\left(\sigma -E\varepsilon \right)}{k_{B}T}\right).$$

Applying logarithm on the latter equation, we obtain the linear relation between $\ln\dot{\varepsilon }$ and strain ε that can be verified in experiments (see Section 3.3), viz.,

(23) $$-\ln\dot{\varepsilon }\;\;=\;\;\ln A_{S}+\frac{E_{0}+b\sigma }{k_{B}T}-\frac{E}{k_{B}T}\varepsilon .$$

The general analytic solution of the KVDE Equation (22) is given by the following logarithmic function of time:

(24) $$\varepsilon \left(t\right)\;\;=\;\;E_{0}+\frac{\sigma }{E}+\frac{k_{B}T}{E}\ln\left(\frac{1}{A_{S}}\frac{Eb(t-t_{0})}{k_{B}T}\right).$$

**Figure 3:**
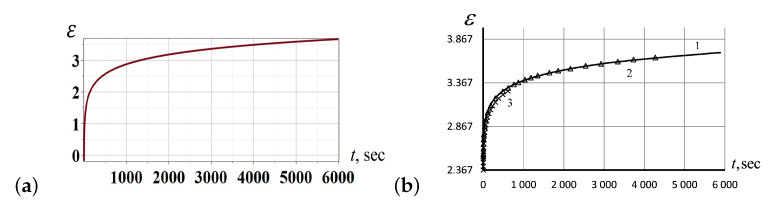
(**a**) A particular solution (24) of the KVDE (22) for $t_{0}=0$. (**b**) The experimental creep curves for silicon rubber under the initial stress $\sigma_{0}=6.4947$ MPa, at the temperatures $-80.3{\;}^{{}^{\circ}}$C (solid curve 1), $-80.6{\;}^{{}^{\circ}}$C (triangles, curve 2), and another time at $-80.6{\;}^{{}^{\circ}}$C (crosses, curve 3) for reproducibility.

In Figure 3a, we show a particular solution curve (24) of the KVDE (22) for $t_{0}=0$. The experimental curves of creeping behavior in silicon rubber were studied experimentally under various loads and temperatures (Figure 3b) matching the analytic solution curve perfectly.

The analytic solution (24) of the KVDE (22) enables a simple prediction of creep curves. In particular, this solution makes it possible to determine the expected time required to reach the maximum creep values for given load and temperature, limiting the exploitation of a product made of rubber. Solving (24) for the time variable, we obtain the following formula for the *remaining useful life* (RUL), estimating the amount of time the product of rubber is likely to operate before it requires repair or replacement, viz.,

(25) $$\left(t-t_{0}\right)\;\;=\;\;A_{S}\frac{k_{B}T}{Eb}\exp\left(-\frac{E(E_{0}-\varepsilon )+\sigma }{k_{B}T}\right).$$

The linear dependence of $\ln\dot{\varepsilon }$ on strain ε makes it possible to predict the time of reaching a scheduled deformation by extrapolation of this linear dependence under constant loads.

## 3. Results: Experimental Verification of Entropic Elastic Forces

In the present section, we report on the experimental verification of the EEF-related effects on non-Newtonian fluids, deforming polymers, and creeping silicon rubber.

### 3.1. Experimental Verification of the Entropic Nature of Viscosity Anomaly

**Figure 4:**
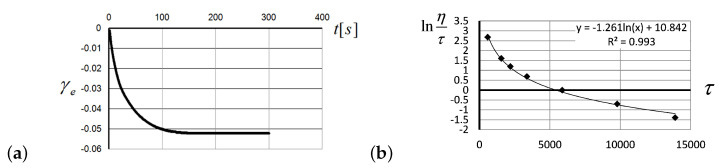
(**a**) The recovery of reversible deformation, $\gamma_{e}$, over time after rotation shut down in polystyrene melts at $190{\;}^{{}^{\circ}}C$, with shear rate $\dot{\gamma }=0.012$ s${}^{-1}$, shear stress τ=114.3 Pa, and the total value of reversible deformation γ=1.135. (**b**) The value of $\ln\left(\eta /\tau \right)$ as a function of share stress τ for polystyrene melts at $180{\;}^{{}^{\circ}}C$, for the stretching rates ranging from 0.0648 s${}^{-1}$ to 2 s${}^{-1}$.

**Figure 5:**
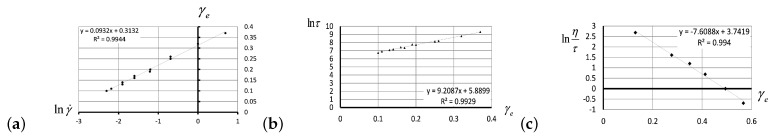
(**a**) The linear relationship between the value of reversible deformation, $\gamma_{e}$, and the logarithm of shear rate, $\ln\dot{\gamma }$, for polystyrene melts at $190{\;}^{{}^{\circ}}C$, measured sequentially at the following shear rates: 0.100 s${}^{-1}$, 0.113 s${}^{-1}$, 0.150 s${}^{-1}$, 0.150 s${}^{-1}$, 0.201 s${}^{-1}$, 0.201 s${}^{-1}$, 0.299 s${}^{-1}$, 0.300 s${}^{-1}$, 0.500 s${}^{-1}$, 0.500 s${}^{-1}$, 1.00 s${}^{-1}$, and 2.00 s${}^{-1}$. (**b**) The linear relationship between the reversible deformation, $\gamma_{e}$, and logarithm of shear stress lnτ for polystyrene melts at $190{\;}^{{}^{\circ}}C$ measured at the constant shear rates, as specified in (**a**). (**c**) The value of $\ln\left(\eta /\tau \right)$ plotted against the volume of reversible deformation, $\gamma_{e}$, measured in polystyrene melts at $180{\;}^{{}^{\circ}}C$, for the stretching rates ranging from 0.0648 s${}^{-1}$ to 2 s${}^{-1}$.

The linear and log-linear relationships predicted by ((6)–(8)) between the logarithms of viscosity η, shear stress τ, and share rate $\dot{\gamma }$ have been confirmed experimentally for the flow of polystyrene, PSC 1540 (AS) Crystal Polystyrene (Total S.A, Paris, France), with a melt flow index 12 g/10 min ($200{\;}^{{}^{\circ}}C$–5 kg, ASTM D1238G), using a Rheometer RHEOTEST^®^RN4 (Rheotest Medingen GmbH, Germany) as a rotational viscometer. The viscosity measurements were carried out using a cone and plate system with the cone diameter 36 mm and angle $5^{{}^{\circ}}$. The temperature during measurements was maintained with an accuracy of $0.1{\;}^{{}^{\circ}}C$. The shear rate was set constant in the range from 0.01 s${}^{-1}$ to 10 s${}^{-1}$. The rotation time before stopping varied from 30 s at high rotation rates to 30 min at low rates of shear deformation. The shear deformation value at a constant shear rate varied from tens to a hundred shear units. After stopping the rotor and its release from external forces, the observation time of backward rotation was typically 300 s. The measurements of viscous flow of melted polymer were carried out at $10{\;}^{{}^{\circ}}C$ intervals, from $180{\;}^{{}^{\circ}}C$ to $220{\;}^{{}^{\circ}}C$. The observed curves are shown in Figure 4 and Figure 5.

For the low shear rates, after the application of default shear stress on melted polystyrene, the reversible deformation of 5.2% was observed after reaching the state of equilibrium recovery (Figure 4a). The logarithmic trend line describing the relation between share stress τ and the value of $\ln\left(\eta /\tau \right)$ in Figure 4b is also a manifestation of EEF, reducing the activation energy of the MKU in the flow of polymer melts.

To verify the relationships (6)–(8), following from a theoretical model, the values of reversible deformation, $\gamma_{e}$, were measured at a constant shear rate, $\dot{\gamma_{e}}$ (Figure 5a); shear stresses τ were measured for the same shear rates (Figure 5b); finally, the value of $\ln\left(\eta /\tau \right)$ was measured as a function of reversible deformation $\gamma_{e}$ (Figure 5c).

All empirically observed patterns presented in Figure 5 have linear trend lines, as predicted by (6)–(8), accounting for the entropic nature of elasticity in polymers. The reported experimental results confirm that the activation energy in polymer melts decreases by an amount proportional to the magnitude of EEF, and by the volume of reversible elastic deformation of molecular chains stretched by the flow. Measuring the slopes of trend lines shown in Figure 5, we can estimate the activation coefficient δ=6.016 kJ/mol = 1.44 kKal/mol, and activation energy $E_{0}=100.92\;$ kJ/mol = 24.1 kKal/mol for polystyrene.

### 3.2. Experimental Verification of the Effect of Elastic Entropic Forces on Elastomer Tensile Curves

Rubber stretching experiments were carried out on a Zwick-Z 010 testing machine (the ZwickRoell Group, ZwickRoell GmbH & Co., KG, Germany) equipped with a MultiXtens extensometer pursuant to DIN EN ISO 527-2/S2. The dumbbell-shaped specimen of a silicone PMVS rubber (ISO 527-2) used in the experiment had rectangular sections that were 2 mm thick, 4 mm wide, with a rectangular narrow section length of 43 mm. The test length of a specimen measured with the extensometer was 15 mm. The molecular weight of a chain segment used to calculate Young’s modulus *E* in (9) was taken as $M_{c}=8\times 10^{4}.$ The average molecular weight for the PMVS rubber specimen was assessed as $M=6\times 10^{5}$.

Hooke’s modulus, H=1793 MPa, was calculated as the limiting modulus for the glassy state of silicon rubber (in accordance with Table II,3 of Tobolsky’s monograph [25]). The series of hysteresis curve measurements were performed repeatedly (through 5 rounds of tensile deformation) at the extension rates of 250 mm/min and 25 mm/min at room temperature. Other series of measurements were performed at $50{\;}^{o}$C (2 series), at $75{\;}^{o}$C (3 series), and at $100{\;}^{o}$C (2 series), respectively—all at an extension rate of 25 mm/min. Deformation of specimens was terminated at the stress reading of ca. 3.8 MPa. Then, each tensile strain was immediately followed by a reverse compressive strain at a contraction rate of the specimen equal to the tensile rate used before, until the stress reading returned to its original value of 0.1 MPa, after which the specimen was immediately stretched again.

**Figure 6:**
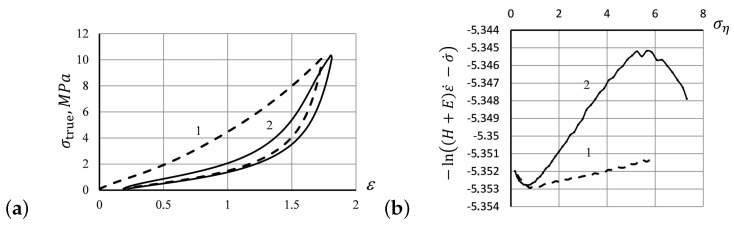
(**a**) True stress vs. strain dependence for silicone rubber at room temperature at a tensile rate of 250 mm/min for the first (dashed line 1) and second (solid line 2) hysteresis cycles. (**b**) Dependence of the value −$\ln\left(\left(H+E\right)\dot{\varepsilon }-\dot{\sigma }\right)$ vs. $\sigma_{\eta }\equiv \left(\sigma -E\varepsilon \right)$ for the ascending strain curve shoulder of the first hysteresis cycle (dashed line 1) and the second hysteresis cycle (solid line 2).

The dependence of true stress, taking into account the change in cross-sectional area of the specimen as it is stretched versus applied strain, is shown in Figure 6a for the first (1) and second (2) hysteresis loops, shown in Figure 1a. In Figure 6b, a crossover behavior is observed in the tensile curve, as the stress exceeds $\sigma_{\eta }=0.714$ (which corresponds to the strain values over ε=20%): a decrease in the rate of stress growth with increasing albeit small strains is flipped to a rapidly accelerating stress for stronger strains. A possible explanation for the observed crossover phenomenon is that the plastic flow characterized by a decrease in the activation energy at small deformations of the material gets overturned by the deformation resistance of the main elastomer network when the EEF comes into play as the tensile process continues [30].

The linear dependence between the values of $-\ln\left(\left(H+E\right)\dot{\varepsilon }-\dot{\sigma }\right)$ and $\sigma_{\eta }$ as predicted in (16) in the framework of the modified SLS model, for the ascending and descending segments of hysteresis curves, allows for estimating Flory’s correction factor, *g*, calculated over the maximum correlation of linear regression (with the coefficient of determination $R^{2}$) in the observed stress versus strain dependence data. The derived assessments of Flory’s correction factor for the different strain intervals are summarized in Table 1 for the linear segments of increasing deformations of the first hysteresis cycle (dashed line) and the second hysteresis cycle (solid line). Table 1 also represents the coefficient of determination $R^{2}$, which measures how well the proposed linear regression model fits the data. In Table 2, we show the same data for the experimental reverse strain curves describing the first (1) and second (2) hysteresis cycles.

**Figure 7:**
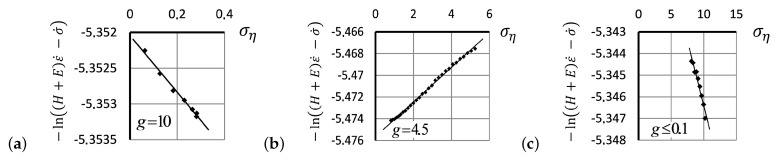
Approximation of the second hysteresis loop in the framework of linear relations (16) in the framework of the modified SLS model requires three linear segments: the initial (**a**), main (**b**), and final (**c**) segments of the ascending tensile curves.

A gradual *decrease* of structure resistance in a response to sequentially applied external stress is observed in repeating hysteresis cycles. The decreasing of structural resistance over the deformation process manifests itself in the emergence of a *third* segment in the ascending tensile curves shown in Figure 6a. Correspondingly, three linear segments—the initial, main, and final—approximating the second hysteresis round are shown in Figure 7a–c. For calculating the modulus *E*, the selection of correction factor *g* was made using the maximum value of the correlation coefficient between the linear relation predicted in (16).

The subsequent hysteresis loops are deformed increasingly, round after round. More linear segments following the relations (16), with different values of Flory’s correction factors, may be introduced for the reliable approximation of experimental tensile curves.

### 3.3. Experimental Study of Creeping Behavior in Silicon Rubber

**Figure 8:**
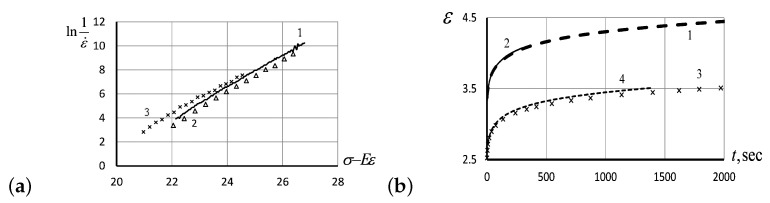
(**a**) The creep curves (PMVS) and their approximation according to the KV model (22) taken for an initial stress of 6.4947 MPa. (**b**) The creep curves (PMVS) observed at the value of initial stress 8.1633 MPa, at temperatures of $26.2{\;}^{{}^{\circ}}$C (curve 1) and $54.8{\;}^{{}^{\circ}}$C (curve 2), as well as at the initial stress value of 6.495 MPa at temperatures of $80.3{\;}^{{}^{\circ}}$C (curve 3) and $106.5{\;}^{{}^{\circ}}$C (curve 4).

The creeping behavior in silicone rubber was studied on a testing stand equipped with a mechanism to load a silicon rubber (PMVS) specimen smoothly at a given rate, in a testing chamber with temperatures ranging from room temperature up to $120{\;}^{{}^{\circ}}$C. Deformation in silicon rubber was measured with the help of a mechanical optoelectronic device, with an accuracy of 0.39 mm and the total deformation of up to 300 mm. Tested specimens were cut out from a 2 mm thick rubber plate, herewith the working part of the specimen had a length of 43 mm and width of 3 mm. The initial stress $\sigma_{0}$ applied to a rubber specimen was calculated by dividing the load by the cross-sectional area of the specimen before loading. The creep curves measured under the same loads for three different time periods are exemplified in Figure 8b for the creep curves taken at different temperatures and initial stress values, as specified in the figure caption.

In Figure 8a, we have presented the PMVS creep curves and their approximation according to the KV model (22) at an initial stress of 6.4947 MPa. As seen from the plots in Figure 8b, showing the dependence of deformation over time, the creep curves coincide for the same values of initial stress, for two initial stresses tested, as well as at four different temperatures when two temperature values were tested for each load.

**Figure 9:**
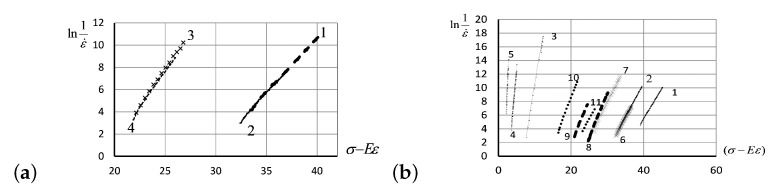
(**a**) Approximation of the creep curves shown in Figure 8b in the framework of the KV model (22). The descriptions of the lines are the same as in Figure 8b. (**b**) Linearity of the $-\ln\dot{\varepsilon }$ value against (σ−Eε) as predicted by the KV model (22) has been verified for PMVS rubber, for a variety of initial stresses and at various temperatures: at $25{\;}^{{}^{\circ}}$C (shown by solid lines), for the following initial stress values—8.835 MPa (curve 1), 8.163 MPa (curve 2), 3.365 MPa (curve 3), 2.428 MPa (curve 4), and 1.722 MPa (curve 5); at $55{\;}^{{}^{\circ}}$C (shown by crosses), for the following initial stress values—8.163 MPa (curve 6) and 7.228 MPa (curve 7); at $55{\;}^{{}^{\circ}}$C (shown by dashed lines): 7.037 MPa (curve 8) and 6.495 MPa (curve 9); at $100{\;}^{{}^{\circ}}$C (shown by points)—5.679 MPa (curve 10) and 6.909 MPa (curve 11).

The creep curves shown in Figure 8b have been calculated theoretically for the specified data (for two different values of stresses, at four different temperatures for each stress value), in the framework of the KV model (22). The theoretical results were juxtaposed with the empirical measurements in Figure 9a, showing a perfect match. The latter graph demonstrates convincingly that the linear relation between $-\ln\dot{\varepsilon }$ and (σ−Eε) does exist, as predicted by the KV model (22). The linear relation following from the KV model has been verified extensively, for the different values of initial stress and at various temperatures (see Figure 9b). All experimental observations justify the applicability of the KV model based on Eyring’s concept of the influence of stresses on the deformation activation energy and show the reliability of theoretical predictions for a wide range of initial stresses and temperatures in silicon rubber.

**Figure 10:**
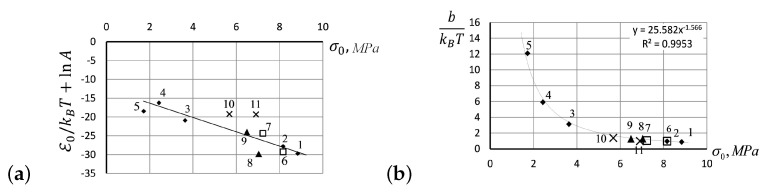
(**a**) The slopes of creep curves, $\ln\eta_{0}=\ln A+E_{0}/k_{B}T,$ shown in Figure 9b against the values of initial stress. (**b**) The viscous volume coefficient $b/k_{B}T$ shown against the values of initial stress $\sigma_{0},$ with the stress and temperature data specified in Figure 9b.

The slopes of creep curves, $\ln\eta_{0}=\ln A+E_{0}/k_{B}T,$ shown in Figure 9b appear close to each other if taken at the same initial stress, even though there were significant temperature differences. To verify the dependence of these slopes upon the values of initial stress applied to the rubber specimen, in Figure 10a, we have shown the values of slopes, against the values of applied stress, $\sigma_{0}$. Accordingly, the data about stress and temperature are given in the caption of Figure 9b. Analogously, the viscous volume coefficient $b/k_{B}T$ depends upon the initial stress values $\sigma_{0}$, as shown in Figure 10b.

**Figure 11:**
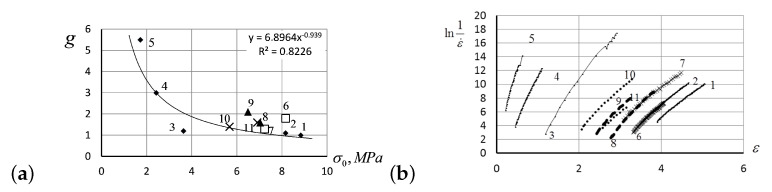
(**a**) Values of Flory’s correction factor *g* against the initial stress values $\sigma_{0}$ in the measurements with stress and temperature specified in Figure 9b. (**b**) The values of $-\ln\dot{\varepsilon }$ against strain ε measured at a variety of initial stress values and at various temperatures. Solid lines are for measurements taken at $25{\;}^{{}^{\circ}}$C, for the following stress values: 8.835 MPa (curve 1), 8.163 MPa (curve 2), 3.365 MPa (curve 3), 2.428 MPa (curve 4), and 1.722 MPa (curve 5). Crosses show the measurements taken at $55{\;}^{{}^{\circ}}$C, for the following stress values: 8.163 MPa (curve 6) and 7.228 MPa (curve 7). Dashed lines show the measurements taken at $80{\;}^{{}^{\circ}}$C, for the following stress values: 7.037 MPa (curve 8) and 6.495 MPa (curve 9). Points stay for the measurements taken at $100{\;}^{{}^{\circ}}$C, for the following stress values: 5.679 MPa (curve 10) and 6.909 MPa (curve 11).

In Figure 11a, the values of Flory’s correction factor, *g*, for the creep curves with initial stresses from 1.7275 MPa to 8.635 MPa at four various temperatures are shown. In our experiments, the Flory factors, reflecting the reaction of current physical and chemical structure of the material to the particular conditions of the creep process, did not depend on creep temperature, but instead depended on initial stress: *g* has high values at low initial stress, but low at high stress. Apparently, a large number of physical cross-links characterized by long relaxation times are not destroyed during deformation under low loads in elastomers. In Figure 11b, we present the experimentally observed linear relations between $\ln\dot{\varepsilon }$ versus strain ε, for creep curves at the different values of temperature and initial stress as specified in the figure caption.

## 4. Discussion

From the analysis of experimental data reported in Section 3 on the hysteresis curves (Section 3.2) and the calculations of Flory’s correction factors (Section 3.3), the following conclusions can be drawn:

1. The experimentally recorded values of the Flory correction coefficient *g* depend on neither temperature nor stretching rate. We, therefore, assume that the value of *g* may characterize the tendency of polymers to maintain a stable structure in mechanical deformation.
2. Flory’s corrections measured for the repeated hysteresis loops were close to each other. Only the first hysteresis round seems to differ substantially from the others, as also predicted by the analytic solutions of the model Equations (18)–(20). The prominent distinction between the first stretching of the specimen and the subsequent rounds indicates an irreversible change that occurs in the polymer structure due to the rupture of weak structural constituents, after which the system acquires a more deformation resistible structure, as manifested by the Mullins effect. The specimen acquires a slight residual deformation, which changes a little during subsequent hysteresis cycles (see Figure 1a)
3. Up to five stable segments can be identified visually on the experimental hysteresis curves. In particular, there are three regions of increasing deformation and two regions of reversible deformation. The measured values of Flory corrections exhibited sufficient reproducibility for all tested samples. For the tested PMVA specimen, the recorded Flory correction factor was 5–7 units.
4. A quantitative description of elastomer deformation can be obtained, using the basic equations of the statistical theory of rubber elasticity and Eyring’s equation modified to take into account the entropic nature of deformation in polymers. For the stretching phase of rubber deformation, the elastic forces increase the activation energy, while they decrease during the retraction deformation.
5. The small segments at the beginning and at the end of tensile curves (denoted as the initial and final segments of hysteresis cycles) show a stress growth slowdown, which may be associated with a decrease in the activation energy. There was a significant change in the values of Flory corrections in these segments at the same time. The final section of the return hysteresis curve has a particularly sharp increase in the Flory correction factor. This can be interpreted as a result of a strong increase in the number of physical cross-links at the final stage of the elastomer chain folding process.

The elastomer molecular structure is a major factor influencing the deformation behavior, as it determines how much and how quickly stress increases, as well as how strong the macromolecules would resist the external force when stretched. The molecular structure of a network formed by chemical and physical bonds changes under tensile and compressive strain, forming a reproducible pattern of deformation behavior that is consistently repeated in a series of measurements of the hysteresis loops in elastomer. The sequential hysteresis rounds are expressed in the consecutive decrease and increase of the jump activation energy required for the migration of MKU into vacancies over material deformation. The changes in energy barriers manifest an increase and decrease in the viscosity of the material during stretching and plastic flow. The anomalous viscosity affects the slowing down and acceleration of stress growth observed in the stretching and contraction curves measured in elastomers.

We profoundly thank our reviewer for the inspiring questions about extending our model to branched macromolecules. One of us (V.K.) considered the possibility of evaluating the rheological behavior of melts of linear polymers under the influence of EEFs on the activation energy decrease during the folding of macromolecules in the course of plastic flow. This process is easily observable when the rheometer’s rotor stops, i.e., when it is free from the engine and rotates backward due to the reversing flow of the polymer melt [14]. In this case, we observed a decrease in viscosity with an increase in shear rate or stress, i.e., the well-known viscosity anomaly.

**Figure 12:**
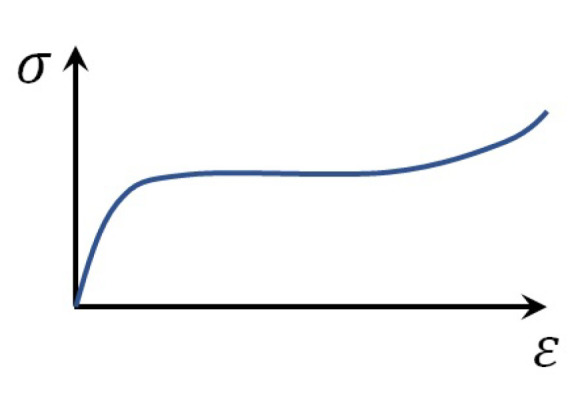
A schematic representation of a possible tensile curve for stretching branched polymers.

For branched (but not chemically cross-linked) polymers, it would be very interesting to study the flow processes in rheometers. We would expect to observe a decrease in viscosity with increasing flow speed or shear stress. However, due to the action of physical cross-links between the entangling sufficiently long branches of chains that are associated with the main chain, the possible deviations towards increasing viscosity at the flow onset, as well as at the end of the flow, should be observed. When stretching branched polymers at a given rate on tensile machines, the dependence of stress on strain should have a large plastic flow region, with growing initial and final sections of the stretching curve (as exemplified schematically by a schematic curve shown in Figure 12).

A similar graph is shown in a recent article [46] kindly pointed to us by our reviewer. In the same article, an increase in viscosity with increasing flow time is indicated. This may indicate that branching promotes the formation of physical cross-links, increasing the resistance to flow instead of decreasing the value of activation energy.

It is worth mentioning that the applicability of molecular models to the flow processes in polymer melts is wide, including not only measuring the properties of flows in rheometers, but also in capillary viscometers, including simplified measurements of the melt flow index (MFI). Instruments for MFI measurements are often used by industry technologists. Moreover, this approach can also be used for measuring the viscosity of melts of the rod extruded from the production extruder when obtaining polymer granules.

Further research is needed to focus on possible applications of the molecular models to the MFI measurements.

We also profoundly thank another reviewer for pointing us at the potential problem of irreversible entropy production (IEP) [47] in polymer deformation. It is worth mentioning that the viscous flows and deformation of polymers exhibit *reversible* properties under mechanical stress (pertaining to zero entropy production), in contrast to low-molecular solids and liquids with an atomic or low-molecular structure. However, polymers, of course, exhibit irreversible deformation as well, resulting in violation of their initial structure. Therefore, the irreversible deformation processes discussed in our work should be intimately tied with the IEP processes, although a unifying theory of entropy production that is valid for general thermodynamic processes, especially describing deformations in different media, has not yet been formulated [47]. Indeed, the formulation of such an entropy production problem *cannot be universal*, as it ultimately depends on the underlying physical system and its governing dynamical laws [47]. Present works on the IEP in the course of deformations (in metals) are purely theoretical [48] and do not concern any experimental verification. Our present work is centered around *interpreting ongoing experimental observations* in the framework of experimentally mastered statistical mechanics focused at possible corrections close to equilibrium—and it is a natural “constructive” limitation of our approach, indeed.

*Further decades* of intensive experimental research will be needed to adopt the relatively novel theoretical concepts of irreversible entropy production [49,50] to the realm of experimentally measurable quantities demanded by industry.

## 5. Conclusions

In our work, we study the relations between the mechanical properties of elastomers and their molecular network structure. Based on the basic equations of the classical statistical theory of rubber elasticity, we have shown that it is possible to quantify the effects of labile physical bonds on the deformation behavior in elastomers.

The role of physical bonds in rubber deformations was evaluated based on the Flory correction factor, which takes into account how bonding defects in polymers can affect the course of the deformation process in hysteresis.

The use of Eyring’s equation, considered in the spirit of Frenkel’s ideas about the vacancy mechanism of flow in condensed media, made it possible to eliminate the primary inconsistency found in the previous quantitative descriptions of tensile curves for rubber, especially at large strains. The conceptual improvement was achieved by modifying the exponential viscous flow equation for polymers to take into account the decrease or increase in the activation energy of deformation by an amount proportional to the entropic elasticity force excited by macromolecule structures subjected to tensile stress.

When macromolecular materials, such as synthetic polymers, natural plants, or other biological materials are exposed to external stress, the entropic nature of viscosity should always be taken into account as follows:

(26) $$\eta \;\;=\;\;Af_{e}\exp\frac{E_{0}\pm bf_{e}}{k_{B}T}$$

where η is the flow viscosity coefficient, $f_{e}$ is the magnitude of the EEF, $E_{0}$ is the activation energy required to overcome the jump potential barrier, and finally, *A* and *b* are the parameters calculated in accordance with the theory of Eyring et al.

A modified SLS model was used for quantifying the stress–strain dependence and calculating the isothermal Young’s modulus. The value of Flory’s correction factor was included in the equations as a measure of the number of physical bonds that influences the deformation behavior in elastomers. The study of consecutive hysteresis loops revealed distinctive, consistently repeating segmentation patterns in the deformation behavior of silicone rubber. The data obtained showed good reproducibility for the numerical values of Flory corrections proportional to the number of possible physical cross-links present in reticulated elastomeric materials when they are subjected to mechanical stress.

Our study suggests that the network structure of polymers is coherent with their mechanical properties and deformation mechanisms.

## Figures and Tables

**Table 1:** Flory’s correction factor *g* estimated over the linear segments of hysteresis curves as predicted in (16), in the framework of the modified SLS model, for the stretching deformation in hysteresis cycles shown in Figure 6a.

Hysteresis Cycle	Ascending Strain Interval	*g*	Determination, $R^{2}$	Tensile Slope, $b/k_{B}T$
1	ε∈ [1.8–20%]	24	0.9956	−0.0037
1	ε∈ [21–170%]	0.6	0.9960	+0.0003
2	ε∈ [21–56%]	10	0.9907	−0.004
2	ε∈ [65–164%]	4.5	0.9975	+0.0019
2	ε∈ [166–179%]	0.1	0.9512	−0.0012

**Table 2:** Flory’s correction factor *g* estimated over the main and final descending segments of the reverse strain curves in hysteresis cycles shown in Figure 6a.

Hysteresis Cycle	Ascending Strain Interval	*g*	Determination, $R^{2}$	Tensile Slope, $b/k_{B}T$
1	ε∈ [173–103%]	7	0.9985	−0.0042
1	ε∈ [65–25%]	70	0.9993	+0.0005
2	ε∈ [178–81%]	5.65	0.9971	−0.0038
2	ε∈ [70–29%]	81	0.9984	+0.0005

## Data Availability

Not applicable.

## Abbreviations

The following abbreviations are used in this manuscript:

EEF	Elastic Entropic Force
IEP	Irreversible Entropy Production
KVDE	Kelvin–Voigt Differential Equation
KVM	Kelvin–Voigt Model
MFI	Melt Flow Index
MKU	Molecular Kinetic Units
PMVS	Polymethylvinylsiloxane (rubber)
RUL	Remaining Useful Life
SLS	Standard Linear Solid

## Appendix A. Entropic Force of Elasticity

The most likely distance *r* between the ends of a chain of *N* units, in which the length of each unit is *l*, equals to $r\;=\;\sqrt{2/3Nl^{2}}.$ Assuming that all conformations of a free chain are equiprobable, and therefore its entropy $S_{0}$ is maximal, the entropy value of a conformation characterized by the distance between the ends of chain *r* reads as follows [32]:

(A1) $$S\;=S_{0}-\frac{3}{2}\frac{k_{B}r^{2}}{Nl^{2}}.$$

As the distance *r* changes, the following entropic elastic force emerges along the molecular chain:

(A2) $$f_{e}\;\equiv \;-T\frac{dS}{dr}=\frac{3k_{B}T}{2Nl^{2}}r$$

where *T* is temperature.
